# Control of Neglected Tropical Diseases: Integrated Chemotherapy and Beyond

**DOI:** 10.1371/journal.pmed.0030112

**Published:** 2006-01-31

**Authors:** Jürg Utzinger, Don de Savigny

## Abstract

Utzinger and de Savigny discuss the implications of a new policy paper, which argues that the control of "the big three" diseases (HIV, TB, and malaria) should be integrated with control of the neglected tropical diseases.

Peter Hotez and colleagues, in a new analysis in *PLoS Medicine*, throw down a compelling challenge to the global health community to intensify its response to the neglected tropical diseases. The authors discuss opportunities for integrating neglected disease control with interventions against the “big three,” namely, HIV/AIDS, tuberculosis, and malaria [ 1]. They highlight 13 neglected tropical diseases: seven helminthic infections (e.g., schistosomiasis), three vector-borne protozoan infections (e.g., lymphatic filariasis), and three bacterial infections (e.g., trachoma). The main geographic focus of these diseases is sub-Saharan Africa.

In the last decade, the world has witnessed the establishment of numerous global partnerships for health that address some of the grandest challenges, specifically the big three, child and maternal mortality, and micronutrient deficiencies. These new partnerships have succeeded in substantially raising the profiles of those health problems, generating unprecedented financial resources, and improving the technical means to tackle them. The global health partnerships and additional resources provide leverage to attain health- and poverty-related international development targets, such as the Millennium Development Goals.

In sharp contrast, advocacy to increase public awareness of and response to the neglected tropical diseases has been far less evident. This lack of advocacy can be attributed to the diseases' primary impact on the poorest of the poor in rural and deprived urban settings, the underestimation of their public health and economic significance, and the lack of coordinated research and control efforts. How can this unacceptable situation be changed? Hotez and colleagues provide some bold thoughts for a way forward. Building on several recent publications [ 2–5], Hotez et al.'s contribution is the most complete review of the issues to date. It has the potential to bring the neglected tropical diseases into a global health focus with the necessary attention on partnership building to facilitate integration of chemotherapy-based morbidity control.

## Putting Neglected Tropical Diseases on the Political Radar Screen

From global health and development perspectives, two issues in Hotez and colleagues' analysis are worth highlighting. First, the authors have made a laudable attempt, using a diversity of recent data sources, to update the annual number of deaths and the disease burden—expressed in disability-adjusted life years (DALYs) lost—due to the neglected tropical diseases. Their new estimates are 534,000 deaths per year and 56.6 million DALYs lost. These estimates are more than 3-fold higher than those put forward in the annex tables of the World Health Report 2004 (deaths, 143,000; burden, 17.7 million DALYs) [ 6]. Yet, the true burden of the neglected tropical diseases is likely to be much higher. For example, a recent meta-analysis for schistosomiasis suggests that the burden of this disease might be severalfold higher than the previous estimate [ 7]. In addition, no estimates are currently available for several other neglected tropical diseases, such as food-borne trematodiasis, although they affect millions of people, are associated with severe morbidity if left untreated, and are emerging due to exponential growth of aquaculture (inland freshwater fish production) development [ 8, 9].

Polyparasitism is the norm rather than the exception in the developing world

Second, the article stresses the important, albeit largely overlooked, issue of geographic overlap of the neglected tropical diseases, which emphasizes that coinfections are pervasive. Recent cross-sectional surveys confirmed that polyparasitism is the norm rather than the exception in the developing world [ 10–12]. However, surprisingly few studies have made an attempt to investigate underlying risk factors that govern this phenomenon, or to demonstrate associations between multiple bacterial/parasitic infections and health outcomes. Such associations would prompt an interest in deeper deterministic understanding of the pathways from coinfection to comorbidity, and an interest in how to reduce disease risk.

## Crosstalk between Neglected Tropical Diseases and the Big Three

Hotez and colleagues summarize an emerging body of literature that emphasizes the crosstalk between the neglected tropical diseases and the big three. For example, recent studies suggest that helminthic infections may be associated with an elevated incidence rate of malaria, and may worsen clinical outcomes of malaria. Although this topic is in its infancy, there is a pressing need to further our understanding of this kind of interaction. At a fundamental level, polyparasitisms represent a major opportunity for a deeper understanding of the physiology of homeostatic balance of a host organism harboring multiple bacterial and/or parasitic infections concurrently. At a more pragmatic level, it has been suggested that regular deworming might have ancillary benefits for malaria control and vaccine efficacies [ 2, 13].

## The Proposal: Integrated Chemotherapy

It makes sense to integrate interventions for multiple diseases when those interventions share a common technical approach, a common target population, and a collectively high disease burden. Integrating interventions against multiple diseases is the basis of the cost-effective success of the Expanded Program on Immunization, and, more recently, of the Integrated Management of Childhood Illness [ 14]. Hotez and colleagues propose a pro-poor health policy and strategy that exploits a similar approach to integration. They suggest large-scale deployment of just four safe, efficacious, and orally administered drugs, i.e., albendazole, azithromycin, ivermectin, and praziquantel, which will target over 90% of the neglected disease burden and will result in additional health benefits. Further support for this integrated chemotherapy approach is already in place through donations of the required drugs by major pharmaceutical companies or procurement of the drugs at relatively low costs.

Of course, there are many tactical issues to be addressed concerning this strategy of integrated chemotherapy-based control. What are the most efficient and cost-effective means to rapidly identify or map areas at highest risk of coinfections? Will the approach be effective across a diversity of epidemiological and sociocultural settings? How do we tailor integrated chemotherapy for specific age groups? What are the most appropriate time intervals, delivery systems, and monitoring and evaluation systems, especially for drug interactions, compliance, and development and spread of resistance? How do we move from morbidity control to transmission containment?

## The Way Forward: Thinking Beyond Integrated Chemotherapy

Failure to address this collective burden of disease, now that an integrated approach seems feasible, will result in it moving steadily up the ranking into the top three once the impact of current global health partnerships starts to register. We will have to deal with the burden sooner or later, so why not now so this effort can take advantage of the economies of scale that occur during national scale up of responses to the big three? Hotez and colleagues have made a strong case to act, and have sketched a strategic approach. Now an agenda needs to be developed to map out the way forward. Such an agenda will need to wrestle not just with the challenge of integrating interventions into an appropriately designed package but also with integrating that package into appropriate delivery systems. A growing realization in the context of how the health-related Millennium Development Goals can be achieved, especially in sub-Saharan Africa, is the need for strengthening health systems [ 15–18]. The January 2006 Stockholm meeting, “Malaria and Neglected Tropical Diseases Quick-Impact Initiative,” could serve as a forum for discussions on how to take Hotez and colleagues' proposal forward. The discussions will need to bear in mind the guiding principles stemming from last year's Paris Declaration on Aid Effectiveness ( http://www.oecd.org/dataoecd/11/41/34428351.pdf) and the High-Level Forum on the Health Millennium Development Goals to work toward systemic approaches that are aligned and harmonized with locally owned national programs, and thereby point the way for truly integrated and more sustainable solutions in the years that lie ahead. 3

## Abbreviation

DALY: disability-adjusted life year
